# Prevalence and incidence of diabetic retinopathy (DR) in the UK population of Gloucestershire

**DOI:** 10.1111/aos.14927

**Published:** 2021-06-28

**Authors:** Peter H. Scanlon, Clareece R. Nevill, Irene M. Stratton, Sonia S. Maruti, Elvira L. Massó‐González, Sobha Sivaprasad, Clare Bailey, Michael Ehrlich, Victor Chong

**Affiliations:** ^1^ Gloucestershire Retinal Research Group Cheltenham General Hospital Cheltenham UK; ^2^ Nuffield Department of Clinical Neuroscience University of Oxford Oxford England; ^3^ University of Gloucestershire Cheltenham England; ^4^ Boehringer Ingelheim Pharmaceuticals, Inc. Ridgefield CT USA; ^5^ Boehringer Ingelheim International GmBH Ingelheim Germany; ^6^ Moorfields Eye Hospital London UK; ^7^ Bristol Eye Hospital Bristol UK

**Keywords:** diabetic retinopathy, epidemiology, retinal screening, imaging, sight‐threatening diabetic retinopathy

## Abstract

**Purpose:**

To estimate prevalence and incidence of diabetic retinopathy (DR) in a UK region by severity between 2012 and 2016 and risk factors for progression to proliferative DR (PDR).

**Methods:**

Electronic medical records from people with diabetes (PWD) ≥18 years seen at the Gloucestershire Diabetic Eye Screening Programme (GDESP) and the hospital eye clinic were analysed (HEC). Prevalence and incidence of DR per 100 PWD (%) by calendar year, grade and diabetes type were estimated using log‐linear regression. Progression to PDR and associated risk factors were estimated using parametric survival analyses.

**Results:**

Across the study period, 35 873 PWD had at least one DR assessment. They were aged 66 (56–75) years (median (interquartile range)), 57% male, 5 (1–10) years since diabetes diagnosis, 93% Type 2 diabetes. Prevalence of DR decreased from 38.9% (95% CI: 38.1%, 39.8%) in 2012 to 36.6% (95% CI: 35.9%, 37.3%) in 2016 (p < 0.001). Incidence of any DR decreased from 10.9% (95% CI: 10.4%, 11.5%) in 2013 to 8.5% (95% CI: 8.1%, 9.0%) in 2016 (p < 0.001). Prevalence of PDR decreased from 3.5% (95% CI: 3.3%, 3.8%) in 2012 to 3.1% (95% CI 2.9%, 3.3%) in 2016 (p = 0.008). Incidence of PDR did not change over time. HbA_1c_ and bilateral moderate–severe NPDR were statistically significant risk factors associated with progression to PDR.

**Conclusions:**

Incidence and prevalence of DR decreased between 2012 and 2016 in this well‐characterized population of the UK.

## Introduction

In England and Wales, 3.54 million people (7% of the population) were registered with diabetes in 2018–2019 (NDA 2020).

Diabetic retinopathy (DR) is a microvascular complication of diabetes which remains a common cause of blindness and vision loss among people of working age in the UK (Quartilho et al. 2016).

It is important that epidemiological data on DR are regularly updated as the numbers of people with diabetes increase and the control of modifiable risk factors improves. Many people quote epidemiological data from the Wisconsin Epidemiological Studies (Klein et al., 1984a, 1984b) which commenced using a stratified sample in southern Wisconsin in 1980. In this population, 22.5% of the younger age group <30 and 8.5% of those ≥30 years had proliferative DR (PDR). Sincethen, major international studies of therapies for Type 1 and Type 2 diabetes (DCCT 1995; UKPDS 1998a, 1998b) have led to treatment guidelines for HbA_1c_ and blood pressure, which have impacted the complications of diabetes. Life expectancy for people with diabetes has increased (Miller et al. 2012). This may partly explain the lower prevalence of PDR (10.6%) reported in a more recent UK hospital‐based study (Keenan TD^2013^). There are several reports based on screening programmes (Younis et al. 2003; Younis et al. 2003; Jones et al. 2012; Thomas et al. 2012) in the UK but these do not reflect the whole population as they may not include data from people who may have been referred to the Hospital Eye Clinic.

This study aims to estimate the prevalence and incidence of DR over time by severity and to estimate progression to PDR in Gloucestershire, a county in the South West of England. Gloucestershire has a population of 600 000 people and is served by a countywide Gloucestershire Diabetic Eye Screening Programme (GDESP) and a referral ophthalmology department. These provide ophthalmology care to 33 000 people with diabetes (PWD). Gloucestershire Diabetic Eye Screening Programme (GDESP) maintains a comprehensive register of all people with diabetes in the county, and data are obtained electronically from Primary Care (GP) registers. This database is linked to the pathology laboratories and blood results, including HbA1c. Since 1998, GDESP has offered annual two 45‐degree field mydriatic digital photographic screening to all eligible people with diabetes aged 12 years or above. Those who have had a retinal examination in the Hospital Eye Clinic (HEC) during the previous 12 months are ineligible, and a small number (<1%) are excluded, for example, terminally ill. Attendance is around 80%.

Those found to have referable DR at screening (Scanlon 2017) are referred for further assessment in the HEC. Within the electronic medical record (EMR) system, the DR assessment screen requires the clinician to fill in a structured assessment form based on lesion identification for both new patient and follow‐up appointments.

In Gloucestershire, it is possible to obtain prevalence and incidence figures of DR levels for a high percentage of the whole population because we have access to all the GDESP annual screening results carried out principally in primary care settings, and HEC run in two main hospitals and 5 small rural hospitals across Gloucestershire.

## Methods

We conducted a retrospective, observational analysis using data from PWD aged 18 years and older in Gloucestershire. The Gloucestershire cohort consisted of patients who had HEC electronic medical records (EMR—Medisoft Limited, Leeds, UK) and the DESP electronic screening medical records (ESMR—OptoMize from Northgate Ltd, Hemel Hempstead, UK). This study was part of a wider study characterizing the incidence and prevalence of levels of DR and macular oedema in the years 2012–2016 and the outcomes of treatment in subsequent years. Further details on the population are in the Statistical Analyses section.

GDESP offers two 45‐degree field mydriatic digital photographic screening to the standards of the English NHS DESP (PHE 2013) to all people with diabetes in Gloucestershire but not under the Hospital Eye Clinic (photographing about 80% of those invited each year). Those under the hospital eye clinic receive a clinical examination or ‘DR structured assessment’ (Keenan et al. 2013). This documents the presence or absence of a specified minimum number of clinical signs of DR and maculopathy for each eye on the HEC electronic medical records (EMR—Medisoft Limited, Leeds, UK). When all mandatory fields are completed, an algorithm in the EMR system calculates the grade of DR and maculopathy according to the Early Treatment of Diabetic Retinopathy Study (ETDRS 1991) and NHS Diabetic Eye Screening Programme (Scanlon 2017) classifications of DR. The EMR’s DR structured assessment module was implemented in Gloucestershire in 2006, and its completion is mandated in medical retina clinics for patients with diabetes.

All images are graded by technician graders using the grading protocol of the English NHS DESP (Scanlon 2017). Internal and external quality assurance processes ensure a high standard of image grading. A minimum qualification (PHE, 2017a) is required for screeners and graders, and evidence of taking the monthly External Quality Assurance Test sets (PHE, 2017b) is also required. Internal quality assurance processes require 10% of images graded with no DR and all those with any DR to be graded by a second grader with arbitration grading for differences in opinion.

The English NHS grading classification and its relationship to ETDRS levels is shown in Table S1. 'Any DR' is defined as the detected presence of any feature(s) of DR including a single microaneurysm (MA) or intra‐retinal haemorrhage in one or both eyes. 'Referable DR' at screening is defined as the presence of any of the retinal features which constitute English NHS Diabetic Eye Screening Programme levels R2 (multiple blot haemorrhage, intra‐retinal microvascular abnormalities (IRMA), venous reduplication or venous beading), R3 (new vessels disc or elsewhere, pre‐retinal or vitreous haemorrhage, pre‐retinal fibrosis or tractional detachment) or M1 (exudate within 1 DD of the centre of the fovea, group of exudates or any MA or haemorrhage within 1 DD of the centre of the fovea with a VA of ≤6/12 or 0.30 logMAR). Those people with poor quality images are referred for examination by slit lamp biomicroscopy.

Pseudonymized data sets were extracted from the Gloucestershire ESMR and EMR for attendances between 1 January 2012 and 31 December 2016. Both were used to provide age, gender, ethnicity, diabetes type, date of diabetes diagnosis, HbA_1c_, visual acuity, treatments received, grading of DR and maculopathy, and recording of features present with the ESMR taken as the primary information source (e.g. diabetes type). Assessment of DR severity was defined by the ETDRS final DR Severity Scale (1991) and the English Screening Programme classifications (Table S1). Gloucestershire Hospitals’ method for measuring HbA_1c_ was by Ion‐Exchange Chromatography up to June 2014 and Affinity Chromatography from then. The Pathology department provided a conversion equation to convert all HbA_1c_ measures to the Affinity Chromatography method. Measures of HbA_1c_ were aligned to DR assessments if they were carried out within 90 days.

### Ethics approval

Ethics approval was granted by the NHS Health Research Authority for this study with IRAS project ID: 236309.

### Statistical analyses

#### Baseline characteristics

Characteristics of the population, by baseline DR severity and analysis cohorts, were summarized using descriptive statistics.

#### Prevalence

Twelve‐month prevalence of diagnosed DR was estimated for calendar years 2012–2016 using log‐linear (Poisson) regression models. The numerator was based on the most severe grade received in either eye for each PWD. The denominator was the number of PWD who attended a DR assessment in the respective year.

#### Incidence

Incidence of any DR, moderate NPDR or worse, and PDR were estimated each calendar year using log‐linear (Poisson) regression models. Incident cases (numerator) were defined by two criteria: (a) first time any DR/moderate NPDR or worse/PDR in at least one eye; (b) had a previous record during the study period showing no DR/mild NPDR or no DR/no PDR in both eyes. Incidence was only estimated for calendar years 2013–2016 because grades in 2012 were counted as a baseline grade for progression to incident grades of different DR severity levels in subsequent years. Patients who were included in the denominators each calendar year were those at risk who met the following criteria: (a) on the GDESP register that year, (b) had one or more DR assessments prior to the respective year, (c) no prior records showed any DR/moderate NPDR or worse/PDR in either eye and (d) had one or more assessments during the respective year. These criteria ensure that once people develop DR they are excluded from the estimation of incidence of DR in subsequent years as they no longer fulfil criterion (c).

Four‐year incidence was calculated in a similar manner but taking the cohort of people who were alive and registered to the local programme for the entire 4‐year period 2013–2016, and had at least one record prior and one record during the four‐year period.

#### Risk factors

Any changes in incidence or prevalence over time were tested by including calendar year into the log‐linear regression models as a continuous variable. Gender, diabetes type, age, time since diagnosis of diabetes and HbA_1c_ were tested for their association with incidence and prevalence by fitting univariable and multivariable regression models (multivariable fitted using forward stepwise selection with the likelihood ratio test). Age and time since diagnosis of diabetes were categorized into 5‐year groups, HbA_1c_ was categorized into 10 mmol/mol groups, and when testing diabetes type only those with T1DM or T2DM were included.

#### Progression

Parametric survival analysis was used to assess time to first appearance of PDR in at least one eye (ETDRS ≥ 61) after developing incident moderate–severe NPDR (ETDRS 43–53) during 2012–2016.

Patients were followed until they were found to have PDR at an appointment, or their last appointment during the study period, whichever came first. The event of first PDR was assumed to have occurred between appointments of no PDR and first PDR (interval censored). Proportion of those that developed PDR after 1 and 3 years was estimated from the survival function (obtained using the EM‐ICM algorithm (Gomez et al. 2009)). Weibull models (univariable and multivariable) were fitted by forward stepwise selection with the likelihood ratio test. Hazard ratios (HR) with 95% CIs were obtained. Continuous variables were measured at the date of incident moderate–severe NPDR. HbA_1c_ was included as mean HbA_1c_ at this time point (a weighted mean of current and previous HbA_1c_ measures where more weight is given to more recent measures). As well as the standard risk factors described above, biochemical measures were available from pathology data extractions. Potential risk factors were included in the analysis if at least two‐thirds of the progression cohort had such data recorded.

Statistical analyses were performed with Stata 16.0. Survival functions were fitted with R 3.6.0 using the ‘Icens’ package (Gentleman & Vandal 2019).

## Results

### Eligible subjects

**Figure 1:**
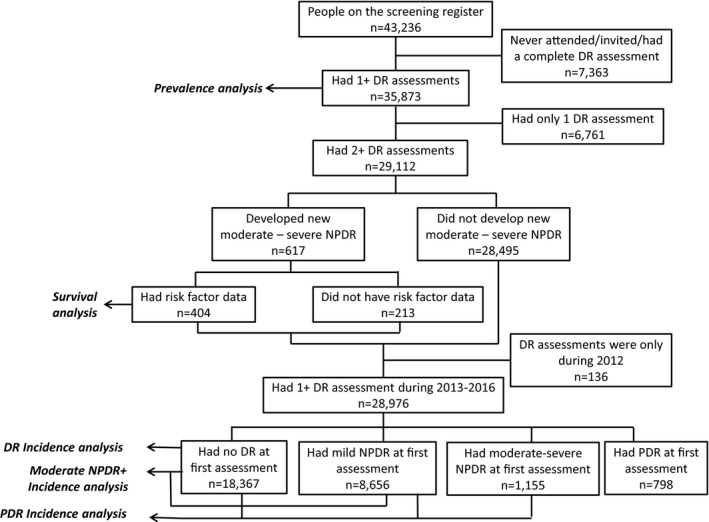
Number of patients for whom data were available for different analyses within the study for the period 1 January 2012 to 3 December 2016. Abbreviations: DR, diabetic retinopathy; NPDR, non‐proliferative DR; PDR, proliferative DR

There were 43 236 Gloucestershire PWD on the screening register during the study period 1 January 2012–31 December 2016, of whom 35 873 (83.0%) PWD had a complete assessment in screening, digital surveillance or the HEC (Figure 1).

### Baseline characteristics

Table 1 shows baseline characteristics of 35 873 PWD with at least one DR assessment.

**Table 1:** Characteristics of the subjects by severity of DR in their worse eye at first DR assessment

Baseline characteristic *n* = 35 873		No DR (*n* = 23 245)	Any DR (*n* = 12 628)	Mild NPDR (*n* = 10 447)	Moderate – severe NPDR (*n* = 1280)	PDR (*n* = 901)
Gender *n* (%*)	Recorded, *n*	23 242	12 628	10 447	1280	901
	Female	10 157 (43.7)	5189 (41.1)	4379 (41.9)	484 (37.8)	326 (36.2)
	Male	13 085 (56.3)	7439 (58.9)	6068 (58.1)	796 (62.2)	575 (63.8)
Age (years)	Median (IQR)	66 (56–75)	65 (54–75)	66 (55–76)	63 (51–73)	60 (48–71)
	Mean (SD)	64.9 (13.7)	63.9 (15.1)	64.7 (15.0)	61.2 (15.2)	58.9 (14.6)
Ethnicity, *n* (%*)	Recorded, *n*	13 160	8828	6689	1253	886
	Caucasian	12 447 (94.6)	8320 (94.2)	6317 (94.4)	1166 (93.1)	837 (94.5)
	Asian	451 (3.4)	293 (3.3)	221 (3.3)	45 (3.6)	27 (3.0)
	Black	145 (1.1)	119 (1.3)	77 (1.2)	29 (2.3)	13 (1.5)
	Mixed	67 (0.5)	66 (0.7)	51 (0.8)	9 (0.7)	6 (0.7)
	Other	50 (0.4)	30 (0.3)	23 (0.3)	4 (0.3)	3 (0.3)
Diabetes Type, *n* (%*)	Recorded, *n*	23 106	12 575	10 406	1278	891
	T1DM	732 (3.2)	1847 (14.7)	1125 (10.8)	317 (24.8)	405 (45.5)
	T2DM	22 361 (96.8)	10 724 (85.3)	9278 (89.2)	960 (75.1)	486 (54.5)
	Other	13 (0.06)	4 (0.03)	3 (0.03)	1 (0.08)	0
Years since diabetes diagnosis**	Median (IQR)	4 (1–7)	9 (4 ‐ 17)	8 (3–14)	16 (11–23)	23 (15–33)
	Mean (SD)	5.0 (5.5)	11.7 (10.6)	10.0 (9.4)	17.5 (10.7)	23.9 (13.3)
Years since T1DM diagnosis**	Median (IQR)	6 (1–12)	23 (15–34)	20 (12–30)	25 (17–34)	32 (23–40)
	Mean (SD)	9.3 (11.3)	24.8 (13.7)	22.1 (13.6)	25.7 (12.0)	31.6 (12.6)
Years since T2DM diagnosis**	Median (IQR)	4 (1–7)	8 (3–14)	7 (2–13)	15 (9–20)	17 (10–24)
	Mean (SD)	4.9 (5.2)	9.5 (8.2)	8.6 (7.5)	14.9 (8.7)	17.6 (10.0)
HbA1c (mmol/mol), *n* (%*)	Recorded, *n*	17 170	9188	7620	918	650
	< 48	5733 (33.4)	1767 (19.2)	1647 (21.6)	67 (7.3)	53 (8.2)
	48‐57	5592 (32.6)	2385 (26.0)	2139 (28.1)	149 (16.2)	97 (14.9)
	58‐85	4652 (27.1)	3805 (41.4)	2999 (39.4)	470 (51.2)	336 (51.7)
	≥ 86	1193 (6.9)	1231 (13.4)	835 (11.0)	232 (25.3)	164 (25.2)
	Median (IQR)	52 (45–63)	59 (50–75)	58 (49–70)	70 (58–86)	71 (59–86)
	Mean (SD)	56.7 (17.0)	64.2 (19.4)	62.3 (18.5)	74.0 (20.7)	73.7 (20.6)
Baseline severity of retinopathy in fellow eye, *n* (%*)	Recorded, *n*	23 245	12 628	10 477	1280	901
	No DR	n/a	n/a	5599 (53.6)	14 (1.1)	12 (1.3)
	Mild NPDR			4848 (46.4)	485 (37.9)	77 (8.5)
	Moderate–severe NPDR			n/a	781 (61.0)	253 (28.1)
	PDR				n/a	559 (62.0)

Baseline was first complete assessment (under HEC, screening or surveillance) within the study period.

Abbreviations: T1DM, *Type 1 diabetes mellitus*; T2DM, *Type 2 diabetes mellitus*; GDESP, *Gloucestershire Diabetic Eye Screening Programme;* DR, *diabetic retinopathy;* NPDR, *non‐proliferative DR;* PDR*, proliferative DR;* HEC*, Hospital Eye Clinic*.

* Percentage was calculated using ‘recorded’ column *n*’s as the denominator

** Date of diagnosis of diabetes was not available for everyone. For 1.5% of people with diabetes, date of registration to the GDESP was used instead.

Of those with moderate–severe NPDR at baseline, 61% had bilateral moderate–severe NPDR. Of those with PDR at baseline, 62% had bilateral PDR.

The incidence analysis of any DR, moderate NPDR or worse, and PDR included 18 367, 27 023 and 28 178 PWD respectively. The survival analysis cohort included 404 PWD. Baseline characteristics of each analysis cohort can be found in Table 2.

### Prevalence

#### Prevalence of diabetes

The unadjusted prevalence of diabetes in Gloucestershire increased from 6.3% (95% CI: 6.2%, 6.4%) per 100 people in 2012 to 6.9 (95% CI: 6.8, 6.9) per 100 people in 2016 (p < 0.001).

Figure S1 indicates higher HbA_1c_ levels in Type 1 than Type 2 patients.

#### Prevalence of DR

Of 35 873 PWD with a record during 2012–2016, 23 245 (64.8%) had no DR, 10 447 (29.1%) had mild NPDR, 1280 (3.6%) had moderate–severe NPDR, and 901 (2.5%) had proliferative DR at baseline.

Analyses of trends over time indicated that the prevalence of any DR per 100 people decreased from 38.9 (95% CI 38.1, 39.8) in 2012 to 36.6 (95% CI: 35.9, 37.3) in 2016 (p < 0.001).

During the same period of 2012–2016, the prevalence of proliferative DR per 100 people decreased from 3.5 (95% CI: 3.3, 3.8) in 2012 to 3.1 (95% CI: 2.9, 3.3) in 2016 (p = 0.008) (Table 2).

**Table 2:** Prevalence of DR by severity, diabetes type and calendar year, per 100 people with diabetes

				2012		2013		2014		2015		2016		IRR (95% CI) for trend over time (increment of calendar year)
Number of Gloucestershire PWD with one or more DR assessments during the respective year (denominator)		Overall		21 487		22 004		22 746		24 967		26 669		
		T1DM		1555		1514		1578		1809		1911		
		T2DM		19 919		20 463		21 086		23 096		24 673		
No DR in both eyes (*n*)				13 118		13 678		14 493		15 925		16 906		
Any DR in at least one eye (ETDRS ≥ 20)	Overall	*n*	8369		8326		8253		9042		9763		0.98 (0.97 to 0.99) p < 0.001	
		Prevalence (95% CI)	38.9 (38.1 to 39.8)		37.8 (37.0 to 38.7)		36.3 (35.5 to 37.1)		36.2 (35.5 to 37.0)		36.6 (35.9 to 37.3)			
	T1DM	*n*	1220		1130		1154		1356		1429		0.99 (0.97 to 1.01) p = 0.319	
		Prevalence (95% CI)	78.5 (74.2 to 83.0)		74.6 (70.4 to 79.1)		73.1 (69.0 to 77.5)		75.0 (71.1 to 79.1)		74.8 (71.0 to 78.8)			
	T2DM	*n*	7139		7182		7079		7664		8312		0.98 (0.98 to 0.99) p < 0.001	
		Prevalence (95% CI)	35.8 (35.0 to 36.7)		35.1 (34.3 to 35.9)		33.6 (32.8 to 34.4)		33.2 (32.4 to 33.9)		33.7 (33.0 to 34.4)			
Mild NPDR in worse eye (ETDRS 20‐35)	Overall	*n* (%a)	6630 (79.2)		6783 (81.5)		6781 (82.2)		7356 (81.4)		8113 (83.1)		0.99 (0.99 to 1.00) p = 0.066	
		Prevalence (95% CI)	30.9 (30.1 to 31.6)		30.8 (30.1 to 31.6)		29.8 (29.1 to 30.5)		29.5 (28.8 to 30.1)		30.4 (29.8 to 31.1)			
	T1DM	*n*	698		652		690		798		862		1.00 (0.98 to 1.03) p = 0.741	
		Prevalence (95% CI)	44.9 (41.7 to 48.3)		43.1 (39.9 to 46.5)		43.7 (40.6 to 47.1)		44.1 (41.2 to 47.3)		45.1 (42.2 to 48.2)			
	T2DM	*n*	5929		6125		6077		6543		7235		0.99 (0.98 to 1.00) p = 0.042	
		Prevalence (95% CI)	29.8 (29.0 to 30.5)		29.9 (29.2 to 30.7)		28.8 (28.1 to 29.6)		28.3 (27.7 to 29.0)		29.3 (28.7 to 30.0)			
Moderate–severe NPDR in worse eye (ETDRS 43‐53)	Overall	*n* (% a)	981 (11.7)		841 (10.1)		809 (9.8)		907 (10.0)		835 (8.6)		0.92 (0.90 to 0.94) p < 0.001	
		Prevalence (95% CI)	4.6 (4.3 to 4.9)		3.8 (3.6 to 4.1)		3.6 (3.3 to 3.8)		3.6 (3.4 to 3.9)		3.1 (2.9 to 3.4)			
	T1DM	*n*	212		174		180		210		195		0.94 (0.90 to 0.99) p = 0.010	
		Prevalence (95% CI)	13.6 (11.9 to 15.6)		11.5 (9.9 to 13.3)		11.4 (9.9 to 13.2)		11.6 (10.1 to 13.3)		10.2 (8.9 to 11.7)			
	T2DM	*n*	767		666		628		695		639		0.92 (0.89 to 0.94) p < 0.001	
		Prevalence (95% CI)	3.9 (3.6 to 4.1)		3.3 (3.0 to 3.5)		3.0 (2.8 to 3.2)		3.0 (2.8 to 3.2)		2.6 (2.4 to 2.8)			
PDR in worse eye (ETDRS ≥ 61)	Overall	*n* (% a)	758 (9.1)		702 (8.4)		663 (8.0)		779 (8.6)		815 (8.3)		0.97 (0.95 to 0.99) p = 0.008	
		Prevalence (95% CI)	3.5 (3.3 to 3.8)		3.2 (3.0 to 3.4)		2.9 (2.7 to 3.1)		3.1 (2.9 to 3.3)		3.1 (2.9 to 3.3)			
	T1DM	*n*	310		304		284		348		372		0.99 (0.96 to 1.03) p = 0.642	
		Prevalence (95% CI)	19.9 (17.8 to 22.3)		20.1 (17.9 to 22.5)		18.0 (16.0 to 20.2)		19.2 (17.3 to 21.4)		19.5 (17.6 to 21.5)			
	T2DM	*n*	443		391		374		426		438		0.95 (0.92 to 0.98) p = 0.002	
		Prevalence (95% CI)	2.2 (2.0 to 2.4)		1.9 (1.7 to 2.1)		1.8 (1.6 to 2.0)		1.8 (1.7 to 2.0)		1.8 (1.6 to 1.9)			

PWD, *people with diabetes*; DR, *diabetic retinopathy*; NPDR, *non‐proliferative* DR; PDR, *proliferative DR*; CI, *confidence interval*; IRR, *incidence rate ratio*; T1DM, *Type 1 diabetes mellitus*; T2DM, Type 2 diabetes *mellitus*.

Prevalence was estimated using Poisson regression, where the denominator was the number of Gloucestershire PWD with at least one assessment during the respective year. For each PWD, their DR severity recorded for the respective year was based on the worst grade given to the worst eye that year. Trend over time was calculated by adding calendar year to the Poisson regression model.

Overall also includes those with ‘other’ and ‘unknown’ type of diabetes.

^a^ Percentage of those with any DR in at least one eye; any DR is the denominator.

**Figure 2:**
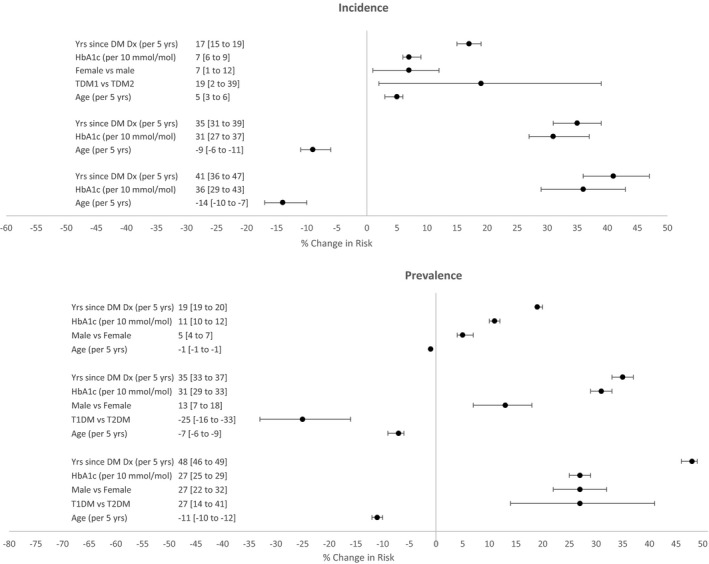
Risk factors from multivariate models of prevalence and incidence of DR, moderate–severe NPDR and proliferative DR. Abbreviations: DR, diabetic retinopathy; PDR, proliferative DR; IRR, incidence rate ratio; CI, confidence interval; T1DM, Type 1 diabetes mellitus; T2DM, Type 2 diabetes mellitus. % Change in risk is calculated from the incidence rate ratios (IRR) from multivariable models ((IRR‐1)*100)

After adjustment for risk factors (Fig. 2) for DR during 2012‐2016, diabetes type was not significantly associated with prevalence of any DR, although, time from diagnosis of diabetes was. T1DM patients were more likely to have higher prevalence of PDR than T2DM patients, but lower prevalence of moderate–severe NPDR.

In the multivariable models, time from diagnosis of diabetes, higher HbA_1c_ levels and being male increased the prevalence of all severities of DR during 2012–2016 with negative associations with age. Results from unadjusted models are in Table S5.

#### Incidence

From 2013 to 2016, 1697 developed new DR in 2013, and then 1447, 1337 and 1374 in subsequent years making a total of 5855 subjects developing new DR.

Furthermore, a total of 606 subjects developed new moderate NPDR or worse, and 269 subjects developed PDR across 2013–2016 (Table 3).

**Table 3:** Incidence of DR each calendar year, overall and split by severity and diabetes type, per 100 Gloucestershire people with diabetes

Number of Gloucestershire PWD with two or more DR assessments where the second or later assessment was *during the respective year/time period*			4‐year [2013‐2016]	2013	2014	2015	2016	IRR (95% CI) for trend over time (increment of calendar year)
			28 976	17 774	20 173	22 372	23 263	
Number with no DR prior (at risk, denominator)		Overall	11 446	15 505	15 341	15 283	16 111	
		T1DM	290	417	446	477	477	
		T2DM	11 154	15 078	14 834	14 736	15 529	
Developed new DR (ETDRS ≥ 20)	Overall	*n*	4612	1697	1447	1337	1374	0.92 (0.90 to 0.94) p<0.001
		Incidence (95% CI)	40.3 (39.1 to 41.5)	10.9 (10.4 to 11.5)	9.4 (9.0 to 9.9)	8.7 (8.3 to 9.2)	8.5 (8.1 to 9.0)	
	T1DM	*n*	151	51	49	60	54	0.99 (0.88 to 1.12) p=0.859
		Incidence (95% CI)	52.1 (44.4 to 61.1)	12.2 (9.3 to 16.1)	11.0 (8.3 to 14.5)	12.4 (9.6 to 16.0)	11.3 (8.7 to 14.8)	
	T2DM	*n*	4461	1646	1398	1277	1319	0.92 (0.90 to 0.94) p<0.001
		Incidence (95% CI)	40.0 (38.8 to 41.2)	10.9 (10.4 to 11.5)	9.4 (8.9 to 9.9)	8.7 (8.2 to 9.2)	8.5 (8.0 to 9.0)	
Number with no DR or mild NPDR prior (at risk, denominator)		Overall	16 401	23 173	24 963	26 378	28 588	
		T1DM	899	1247	1390	1467	1543	
		T2DM	15 499	21 909	23 499	24 817	26 915	
Developed new moderate NPDR or worse (ETDRS ≥ 43)	Overall	*n*	465	147	166	151	142	0.92 (0.85 to 0.98) p=0.015
		Incidence (95% CI)	2.8 (2.6 to 3.1)	0.63 (0.54 to 0.75)	0.66 (0.57 to 0.77)	0.57 (0.49 to 0.67)	0.50 (0.42 to 0.59)	
	T1DM	*n*	85	19	39	28	25	0.96 (0.81 to 1.13) p=0.609
		Incidence (95% CI)	9.5 (7.6 to 11.7)	1.5 (0.97 to 2.4)	2.8 (2.1 to 3.8)	1.9 (1.3 to 2.8)	1.6 (1.1 to 2.4)	
	T2DM	*n*	380	128	127	123	117	0.91 (0.84 to 0.98) p=0.016
		Incidence (95% CI)	2.5 (2.2 to 2.7)	0.58 (0.49 to 0.69)	0.54 (0.45 to 0.64)	0.50 (0.42 to 0.59)	0.43 (0.36 to 0.52)	
Number with no PDR prior (at risk, denominator)		Overall	17 132	24 223	26 110	27 642	29 885	
		T1DM	1092	1494	1654	1770	1853	
		T2DM	16 035	22 710	24 380	25 775	27 900	
Developed new PDR (ETDRS ≥ 61)	Overall	*n*	207	64	57	69	79	1.02 (0.91 to 1.13) p=0.768
		Incidence (95% CI)	1.2 (1.1 to 1.4)	0.26 (0.21 to 0.34)	0.22 (0.17 to 0.28)	0.25 (0.20 to 0.32)	0.26 (0.21 to 0.33)	
	T1DM	*n*	57	18	14	23	27	1.11 (0.91 to 1.36) p=0.284
		Incidence (95% CI)	5.2 (4.0 to 6.8)	1.2 (0.76 to 1.9)	0.85 (0.50 to 1.4)	1.3 (0.86 to 2.0)	1.5 (1.0 to 2.1)	
	T2DM	*n*	150	46	43	46	52	0.98 (0.86 to 1.11) p=0.731
		Incidence (95% CI)	0.94 (0.80 to 1.1)	0.20 (0.15 to 0.27)	0.18 (0.13 to 0.24)	0.18 (0.13 to 0.24)	0.19 (0.14 to 0.24)	

Abbreviations: PWD, *people with diabetes*; DR, *diabetic retinopathy*; PDR, *proliferative DR*; CI, *confidence interval*; IRR, *incidence rate ratio*; T1DM, *Type 1 diabetes mellitus*; T2DM, Type 2 diabetes *mellitus*.

Incidence was estimated using Poisson regression, where the denominator was Gloucestershire PWD at risk. People counted towards the numerator if they had a record of the disease with a previous record of no disease. Trend over time was calculated by adding calendar year to the Poisson regression model.

Incidence could not be estimated for calendar year 2012 due to the requirement of a prior record of no DR and subjects normally being seen annually.

Overall also includes those with ‘other’ and ‘unknown’ type of diabetes.

For 4‐year incidence, subjects had to also be alive, registered and living in the area for the entire 4‐year period to ensure complete follow‐up.

Four‐year incidence of new DR, moderate NPDR or worse, and PDR were 40.3 (95% CI: 39.1, 41.5), 2.8 (95% CI: 2.6, 3.1) and 1.2 (95% CI: 1.1, 1.4) per 100 PWD. Incidence of new DR decreased over time from 10.9% (95% CI: 10.4%, 11.5%) in 2013 to 8.5% (95% CI: 8.1%, 9.0%) in 2016 (p < 0.001). Incidence of moderate NPDR or worse decreased over time from 0.63 (95% CI: 0.54, 0.75) per 100 PWD in 2013 to 0.50 (95% CI: 0.42, 0.59) in 2016 (p = 0.015). Overall, incidence of PDR was not significantly different during 2013‐2016 (p = 0.768) (Table 3). Incidence of PDR per 100 PWD differed by DR severity: four‐year incidence of PDR was 0.31 (95% CI: 0.23, 0.41) among PWD starting with no DR/mild NPDR and 21.3 (95% CI: 18.1, 25.0) among PWD starting with moderate–severe NPDR (Table S6).

Estimation of incidence during 2013–2016 was repeated for T1DM and T2DM separately (Table 3). In T1DM, there was no trend in time seen in incidence across all severities (p = 0.859, p = 0.609, p = 0.284 for any DR, moderate NPDR or worse, and PDR respectively). For those with T2DM, incidence of any DR and moderate NPDR or worse decreased with time (p < 0.001 and p = 0.016 respectively).

The results from multivariable modelling of incidence during 2013–2016 (Fig. 2) show that time since diabetes diagnosis, higher HbA_1c_ levels, older age, being female and having T1DM were associated with higher incidence of any DR. For incidence of moderate NPDR or worse, time since diagnosis of diabetes, HbA_1c_ levels and age were in the final model. For incidence of PDR, time since diagnosis of diabetes, HbA_1c_ levels and age were in the final model.

Results from unadjusted models are in Table S5.

#### Progression to PDR

Of the 404 subjects with incident moderate–severe NPDR in their worse eye (ETDRS level 43–53), 258 (63.9%) had one eye affected and 146 (36.1%) had both affected (bilateral). Of those in whom one eye was affected, 52 developed NPDR in the second eye (median 359 days (IQR: 172–544)).

The estimated survival function of developing PDR from incident moderate–severe NPDR (unilateral or bilateral) indicated that at the end of the 1st year, 4.5% developed PDR and at the end of the 3rd year 13.4% of patients developed PDR. Of those in whom both eyes had NPDR (bilateral) at incident case, 17 patients (12%) went on to develop PDR in at least one eye (median 2.0 years (IQR: 1.2–2.7).

Table S7 shows that, among the 404 patients, those with higher HbA_1c_ and with bilateral moderate–severe NPDR at incident case were more likely to develop PDR. In the multivariable model, both of these were risk factors. Whilst adjusting for other variables, the risk of developing PDR increased by 18% (95% CI: 3%–35%) per 10mmol/mol increase in HbA_1c_ and by 123% (95% CI: 0%–400%) for those with bilateral NPDR versus unilateral.

## Discussion

Whilst there are many studies in the literature describing prevalence and incidence of DR, there are few like Gloucestershire where there is universal health care, free at the point of delivery, with a countywide screening service. Referrals from the screening programme are made to a single eye department. This uses an electronic record enabling reporting of the severity levels of DR, incidence of DR and trends over time. This study covers a high percentage of the population of people with diabetes in the region. We have access to all the screening results and hospital ophthalmology records in the area. Hence, this study is based on a well‐characterized population and is generalizable to the region.

There are important differences between this and earlier studies because the prevalence/incidence results in this study are lower than earlier studies that are commonly reported. This is important for those who are designing studies of medicines to slow down the progression of diabetic retinopathy and those planning clinical services for monitoring and treatment of DR. Very high prevalence levels of DR and PDR were observed in the baseline data from a stratified sample of the population of Wisconsin (Klein et al., 1984a, 1984b) for people with diabetes in 1980. Although considered representative of the population at the time, in group 1 (younger onset people aged <30 years) the prevalence of DR and PDR was 70.7% and 22.5%, in group 2 (aged ≥30 years taking insulin) was 70.1% and 14.1%, and in group 3 (those aged ≥30 years not taking insulin) was 38.7% and 3%. Klein reported the 4‐year incidence figures in 1989 for any DR and PDR in group 1 to be 59% and 10.5%, group 2 to be 47.4% and 7.4%, and group 3 to be 34.4% and 2.3%.

Several landmark studies have led to implementation of guidelines to improve the clinical care of those with diabetes. Randomized clinical trials have shown the importance of glycaemic control (Klein et al., 1989a, 1989b, 1989c; DCCT 1993; UKPDS 1998b; Klein et al. 2009) and control of blood pressure (Klein et al., 1989a, 1989b, 1989c; Joner et al. 1992; UKPDS 1998a; Klein et al. 2009) in the development of STDR and DMO. This is the probable reason why Wong et al. (2009) and Klein et al. (1995) reported lower rates of progression to STDR and visual loss in later time periods.

Although we have previously shown (Scanlon et al. 2015) that the blood pressure (BP) control in our population in Gloucestershire is relatively good, for this study we did not have access to BP results and this is a limitation of our study. This study has shown a link between the prevalence of DR and glycaemic control (Table S5).

We found a decrease in incidence of any DR from 10.9% in 2013 to 8.5% in 2016 (p < 0.001), which may be due to earlier detection of diabetes. Variations in DR levels can occur because of a difference in the stage of detection of diabetes. In the UKPDS study of Type 2 diabetes, the prevalence of any DR and PDR at diagnosis was 36.6% and 0.1%. The incidence of any DR and PDR at 6 years was 22% and 5.6%. Many of these patients were newly diagnosed because they had developed symptoms. In two European populations where screening for diabetes was undertaken, the prevalence of any DR was 6.6% (Bek et al. 2009) and 12% (Ponto et al. 2016) and there were no prevalent cases of PDR. Thomas et al. (2012) reported during 2005–2009 that the annual incidence of any DR in Type 2 diabetes in the screening cohort in Wales decreased from 12.5% in the first year to 6.7% in the fourth year.

Duration of diabetes is known to be a risk factor for DR (Klein et al., 1989a, 1989b, 1989c), which we also found to be a risk factor in the incidence and prevalence of all DR levels (Fig. 2 and Table S5).

Screening studies (Younis et al. 2003; Younis et al. 2003; Jones et al. 2012; Kanjee et al. 2016) have limitations because they refer patients at a level of moderate NPDR or screen‐positive maculopathy and hence do not have accurate data on incidence of PDR. They do, however, have fairly accurate data on any DR. Vujosevic et al. (2017) reported a prevalence of any DR of 27.6% in the screening service that takes place in two diabetes clinics in the area of Padova, Italy. Thomas et al. (2012) recorded a prevalence of any DR in the DR Screening Service for Wales of 30.8% which is comparable with the prevalence of that we found in our study of 36.6% in 2016.

T1DM patients were more likely to have higher prevalence of PDR than T2DM patients and lower prevalence of moderate–severe NPDR. As mean duration of diabetes is far longer in T1DM than in T2DM, duration of diabetes is confounded with type of diabetes. The results from multivariable modelling of incidence during 2013–2016 (Fig. 2) show that T1DM was associated with higher incidence of any DR, whereas, for incidence of PDR, only time since diagnosis of diabetes, HbA_1c_ levels and age were in the final model.

Socio‐economic factors play a part in the development of DR (Varma et al. 2010). Despite the fact that Gloucestershire has relatively few areas of poor socio‐economic status compared to the rest of England, we have previously shown a link (Scanlon et al. 2008) between socio‐economic deprivation and the development of STDR in Gloucestershire, although it is also known (NICE 2011) that socio‐economic factors affect the incidence and development of Type 2 diabetes and this would then have an impact on DR.

Ethnicity plays a part in the development of STDR (Leske et al. 2003; Sivaprasad et al. 2012). The population profile (GCC 2019) showed that the Gloucestershire has a small proportion of people from Black and Minority Ethnic groups accounting for 4.6% of the population.

Thus, we had insufficient heterogeneity to investigate the role of ethnicity in our study.

Most cited studies used photographic methods to record DR levels, the number of fields and field width varying from one 45‐degree field to seven 30‐degree field stereophotography. Moss et al. (1989) demonstrated that the sensitivity of two to four 30‐degree fields compared to seven fields for detecting any DR varies from 87% to 95%. This may have caused small differences in DR levels between studies.

Grading can vary between studies depending on the quality of the grading.

We interpret that the change over time in DR estimates is predominantly a time trend rather than due to ageing. The estimates for incidence and prevalence are likely to have some natural overlap in the denominator each calendar year. Although people ‘dropping out’ from the denominators each year due to ageing and disease progression (for incidence), different people are ‘entering’ the denominator when they join the screening programme. For incidence of any DR, each year 13% were ‘dropping out’ and 15% were ‘entering’ (Table S8). However, as with any other study, there may be an element due to ageing for participants who remained in the denominator across the years.

The principal risk factors that we found for progression of DR in univariable models that did not include current DR levels were duration of diabetes, HbA1c and diabetes type. However, these are not independent of one another (e.g. Fig. S1 shows that the mean HbA1c for people with Type 1 diabetes was higher than those with Type 2 diabetes) and so in multivariable model for progression from incident moderate–severe NPDR to PDR (Table S7), updated mean HbA1c and the presence of moderate–severe NPDR in both eyes were the only significant risk factor.

This study provides epidemiological data on a regional population of the UK. It is important that epidemiological data are regularly updated in populations as the numbers of people with diabetes increase and the control of modifiable risk factors improves.

## Supporting information


**Figure S1**. Mean (95% CI) HbA_1c_ of Gloucestershire people with diabetes on the screening register at diagnosis.

**Table S1**. Diabetic Retinopathy Classifications of Progression to Proliferative DR.

**Table S2**. Baseline characteristics of the subjects within the separate analysis cohorts.

**Table S3**. Prevalence of moderate‐severe NPDR by ETDRS level, diabetes type, and calendar year, per 100 people with diabetes.

**Table S4**. Sensitivity analysis ‐ prevalence of DR by severity and calendar year, per 100 people with diabetes (regardless of appointment attendance).

**Table S5**. Unadjusted risk factors from univariable models of prevalence and incidence of any DR, moderate‐severe NPDR and proliferative DR.

**Table S6**. Incidence of PDR by DR severity (no DR/mild NPDR or moderate‐severe NPDR) and diabetes type, per 100 people with diabetes.

**Table S7**. Risk factors for developing PDR from incident moderate‐severe NPDR (*n patients = 404*).

**Table S8**. Number of participants in the denominator for incidence of any DR each calendar year.

## References

[aos14927-bib-0001] Bek T , Lund‐Andersen H , Hansen AB , Johnsen KB , Sandbaek A & Lauritzen T (2009): The prevalence of diabetic retinopathy in patients with screen‐detected type 2 diabetes in Denmark: the ADDITION study. Acta Ophthalmol 87: 270–274. 18823287 10.1111/j.1755-3768.2008.01207.x

[aos14927-bib-0002] DCCT (1993): The effect of intensive treatment of diabetes on the development and progression of long‐term complications in insulin‐dependent diabetes mellitus. The Diabetes Control and Complications Trial Research Group. N Engl J Med 329: 977–986. 8366922 10.1056/NEJM199309303291401

[aos14927-bib-0003] DCCT (1995): Progression of retinopathy with intensive versus conventional treatment in the Diabetes Control and Complications Trial. Diabetes Control and Complications Trial Research Group. Ophthalmology 102: 647–661. 7724182 10.1016/s0161-6420(95)30973-6

[aos14927-bib-0004] ETDRS (1991): Fundus photographic risk factors for progression of diabetic retinopathy. ETDRS report number 12. Early Treatment Diabetic Retinopathy Study Research Group. Ophthalmology 98: 823–833. 2062515

[aos14927-bib-0005] GCC (2019): Gloucestershire County Council. Population Profile. (Accessed 13/07/20, at https://www.gloucestershire.gov.uk/media/12777/equality‐profile‐2019‐final.pdf.).

[aos14927-bib-0006] Gentleman R & Vandal A . (2019): Icens: NPMLE for Censored and Truncated Data.

[aos14927-bib-0007] Gomez G , Calle ML , Oller R & Langohr K (2009): Tutorial on methods for interval‐censored data and their implementation in R. Statistical Modelling 9: 259–297.

[aos14927-bib-0008] Joner G , Brinchmann‐Hansen O , Torres CG & Hanssen KF (1992): A nationwide cross‐sectional study of retinopathy and microalbuminuria in young Norwegian type 1 (insulin‐dependent) diabetic patients. Diabetologia 35: 1049–1054. 1473614 10.1007/BF02221680

[aos14927-bib-0009] Jones CD , Greenwood RH , Misra A & Bachmann MO (2012): Incidence and progression of diabetic retinopathy during 17 years of a population‐based screening program in England. Diabetes Care 35: 592–596. 22279031 10.2337/dc11-0943 PMC3322726

[aos14927-bib-0010] Kanjee R , Dookeran RI , Mathen MK , Stockl FA & Leicht R (2016): Six‐year prevalence and incidence of diabetic retinopathy and cost‐effectiveness of tele‐ophthalmology in Manitoba. Can J Ophthalmol/Journal Canadien d'Ophtalmologie 51: 467–470. 27938959 10.1016/j.jcjo.2016.05.002

[aos14927-bib-0011] Keenan TDL , Johnston Rl , Donachie P , Sparrow Jm , Stratton Im & Scanlon P (2013): United Kingdom National Ophthalmology Database Study: Diabetic Retinopathy; Report 1: prevalence of centre‐involving diabetic macular oedema and other grades of maculopathy and retinopathy in hospital eye services. Eye London 27: 1397–1404. 10.1038/eye.2013.196 PMC3869516 24051410

[aos14927-bib-0012] Keenan TD , Johnston RL , Donachie PH , Sparrow JM , Stratton IM & Scanlon P (2013): United Kingdom National Ophthalmology Database Study: Diabetic Retinopathy; Report 1: prevalence of centre‐involving diabetic macular oedema and other grades of maculopathy and retinopathy in hospital eye services. Eye (London, England) 27: 1397–1404. 10.1038/eye.2013.196 PMC3869516 24051410

[aos14927-bib-0013] Klein R , Klein BE , Moss SE & Cruickshanks KJ (1995): The Wisconsin Epidemiologic Study of Diabetic Retinopathy. XV. The long‐term incidence of macular edema. Ophthalmology 102: 7–16. 7831044 10.1016/s0161-6420(95)31052-4

[aos14927-bib-0014] Klein R , Klein BE , Moss SE , Davis MD & DeMets DL (1984a): The Wisconsin epidemiologic study of diabetic retinopathy. II. Prevalence and risk of diabetic retinopathy when age at diagnosis is less than 30 years. Arch Ophthalmol 102: 520–526. 6367724 10.1001/archopht.1984.01040030398010

[aos14927-bib-0015] Klein R , Klein BE , Moss SE , Davis MD & DeMets DL (1984b): The Wisconsin epidemiologic study of diabetic retinopathy. III. Prevalence and risk of diabetic retinopathy when age at diagnosis is 30 or more years. Arch Ophthalmol 102: 527–532. 6367725 10.1001/archopht.1984.01040030405011

[aos14927-bib-0016] Klein R , Klein BE , Moss SE , Davis MD & DeMets DL (1989a): The Wisconsin Epidemiologic Study of Diabetic Retinopathy. IX. Four‐year incidence and progression of diabetic retinopathy when age at diagnosis is less than 30 years. Arch Ophthalmol 107: 237–243. 2916977 10.1001/archopht.1989.01070010243030

[aos14927-bib-0017] Klein R , Klein BE , Moss SE , Davis MD & DeMets DL (1989b): The Wisconsin Epidemiologic Study of Diabetic Retinopathy. X. Four‐year incidence and progression of diabetic retinopathy when age at diagnosis is 30 years or more. Arch Ophthalmol 107: 244–249. 2644929 10.1001/archopht.1989.01070010250031

[aos14927-bib-0018] Klein R , Knudtson MD , Lee KE , Gangnon R & Klein BE (2009): The Wisconsin Epidemiologic Study of Diabetic Retinopathy XXIII: the twenty‐five‐year incidence of macular edema in persons with type 1 diabetes. Ophthalmology 116: 497–503. 19167079 10.1016/j.ophtha.2008.10.016 PMC2693093

[aos14927-bib-0019] Klein R , Moss SE , Klein BE , Davis MD & DeMets DL (1989c): The Wisconsin epidemiologic study of diabetic retinopathy. XI. The incidence of macular edema. Ophthalmology 96: 1501–1510. 2587045 10.1016/s0161-6420(89)32699-6

[aos14927-bib-0020] Leske MC , Wu SY , Hennis A , Nemesure B , Hyman L , Schachat A & G Barbados Eye Studies (2003): Incidence of diabetic retinopathy in the Barbados Eye Studies. Ophthalmology 110: 941–947. 12750094 10.1016/S0161-6420(03)00086-1

[aos14927-bib-0021] Miller RG , Secrest AM , Sharma RK , Songer TJ & Orchard TJ (2012): Improvements in the life expectancy of type 1 diabetes: the Pittsburgh Epidemiology of Diabetes Complications study cohort. Diabetes 61: 2987–2992. 22851572 10.2337/db11-1625 PMC3478551

[aos14927-bib-0022] Moss SE , Meuer SM , Klein R , Hubbard LD , Brothers RJ & Klein BE (1989): Are seven standard photographic fields necessary for classification of diabetic retinopathy? Invest Ophthalmol Vis Sci 30: 823–828. 2656572

[aos14927-bib-0023] NDA . (2020): National Diabetes Audit, 2018‐19. Report 1: Care Processes and Treatment Targets. NHS Digital.

[aos14927-bib-0024] NICE . (2011): Socio economic position, the risk of pre and type 2 diabetes, and implications for prevention.

[aos14927-bib-0025] PHE . (2013): Diabetic eye screening programme: pathway for adequate or inadequate images and where images cannot be taken.

[aos14927-bib-0026] PHE (2017a): Continuous Professional Development for Screening ‐ the New Qualification.

[aos14927-bib-0027] PHE . (2017b): Updates to test and training system benefit diabetic eye screening providers.

[aos14927-bib-0028] Ponto KA , Koenig J , Peto T et al. (2016): Prevalence of diabetic retinopathy in screening‐detected diabetes mellitus: results from the Gutenberg Health Study (GHS). Diabetologia 59: 1913–1919. 27314413 10.1007/s00125-016-4013-5

[aos14927-bib-0029] Quartilho A , Simkiss P , Zekite A , Xing W , Wormald R & Bunce C (2016): Leading causes of certifiable visual loss in England and Wales during the year ending 31 March 2013. Eye (London, England) 30: 602–607. 10.1038/eye.2015.288 PMC5108547 26821759

[aos14927-bib-0030] Scanlon PH (2017): The English National Screening Programme for diabetic retinopathy 2003–2016. Acta Diabetol 54: 515–525. 28224275 10.1007/s00592-017-0974-1 PMC5429356

[aos14927-bib-0031] Scanlon PH , Aldington SJ , Leal J , Luengo‐Fernandez R , Oke J , Sivaprasad S , Gazis A & Stratton IM (2015): Development of a cost‐effectiveness model for optimisation of the screening interval in diabetic retinopathy screening. Health Technol Assess 19: 1–116. 10.3310/hta19740 PMC4780979 26384314

[aos14927-bib-0032] Scanlon PH , Carter SC , Foy C , Husband RF , Abbas J & Bachmann MO (2008): Diabetic retinopathy and socioeconomic deprivation in Gloucestershire. J Med Screen 15: 118–121. 18927093 10.1258/jms.2008.008013

[aos14927-bib-0033] Sivaprasad S , Gupta B , Gulliford MC , Dodhia H , Mohamed M , Nagi D & Evans JR (2012): Ethnic variations in the prevalence of diabetic retinopathy in people with diabetes attending screening in the United Kingdom (DRIVE UK). PLoS One 7: e32182. 22412857 10.1371/journal.pone.0032182 PMC3297598

[aos14927-bib-0034] Thomas RL , Dunstan F , Luzio SD , Roy S , Hale SL , North RV , Gibbins RL & Owens DR (2012): Incidence of diabetic retinopathy in people with type 2 diabetes mellitus attending the Diabetic Retinopathy Screening Service for Wales: retrospective analysis. BMJ 344: e874. 22362115 10.1136/bmj.e874 PMC3284424

[aos14927-bib-0035] UKPDS (1998a): Tight blood pressure control and risk of macrovascular and microvascular complications in type 2 diabetes: UKPDS 38. UK Prospective Diabetes Study Group. BMJ 317: 703–713. 9732337 PMC28659

[aos14927-bib-0039] UKPDS (1998b): Intensive blood‐glucose control with sulphonylureas or insulin compared with conventional treatment and risk of complications in patients with type 2 diabetes (UKPDS 33). UK Prospective Diabetes Study (UKPDS) Group. Lancet 352: 837–853. 9742976

[aos14927-bib-0036] Varma R , Choudhury F , Klein R , Chung J , Torres M & Azen SP (2010): Four‐year incidence and progression of diabetic retinopathy and macular edema: the Los Angeles Latino Eye Study. Am J Ophthalmol 149: 752‐761 e751‐753. 20149342 10.1016/j.ajo.2009.11.014 PMC2905589

[aos14927-bib-0037] Vujosevic S , Pucci P , Casciano M et al. (2017): A decade‐long telemedicine screening program for diabetic retinopathy in the north‐east of Italy. J Diabetes Complications 31: 1348–1353. 28551296 10.1016/j.jdiacomp.2017.04.010

[aos14927-bib-0038] Wong TY , Mwamburi M , Klein R et al. (2009): Rates of progression in diabetic retinopathy during different time periods: a systematic review and meta‐analysis. Diabetes Care 32: 2307–2313. 19940227 10.2337/dc09-0615 PMC2782996

[aos14927-bib-0040] Younis N , Broadbent DM , Harding SP & Vora JP (2003): Incidence of sight‐threatening retinopathy in Type 1 diabetes in a systematic screening programme. Diabetic Med 20: 758–765. 12925058 10.1046/j.1464-5491.2003.01035.x

[aos14927-bib-0041] Younis N , Broadbent DM , Vora JP & Harding SP (2003): Incidence of sight‐threatening retinopathy in patients with type 2 diabetes in the Liverpool Diabetic Eye Study: a cohort study. Lancet 361: 195–200. 12547541 10.1016/s0140-6736(03)12267-2

