# Supplementary figures and images for: Prevalence and incidence of diabetic retinopathy (DR) in the UK population of Gloucestershire
Source: Acta Ophthalmol. 2021 Jun 28;100(2):e560–70. doi: 10.1111/aos.14927 (PMC9290830; doi:10.1111/aos.14927)

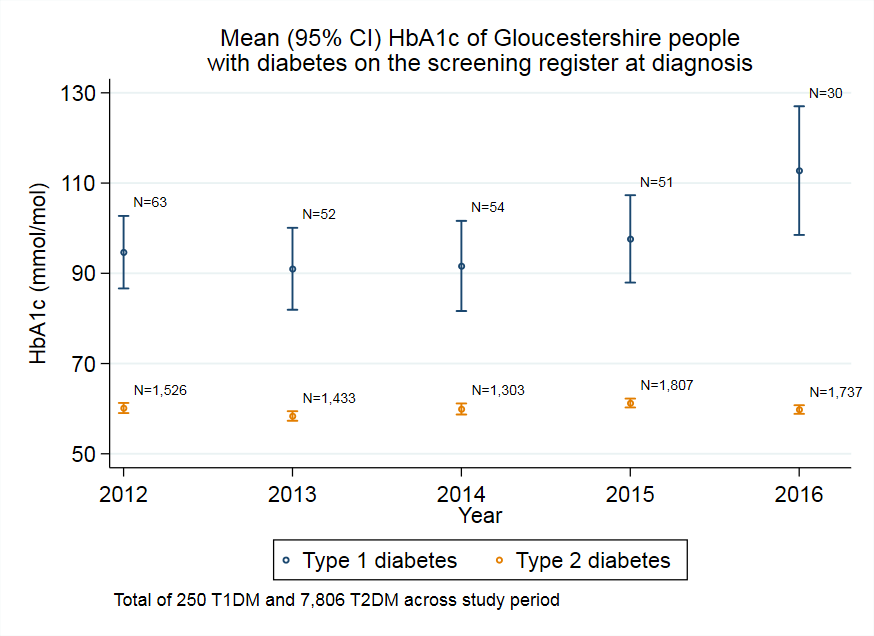

Supplement: Supplementary file 1 — Figure S1. Mean (95% CI) HbA1c of Gloucestershire people with diabetes on the screening register at diagnosis. [file AOS-100-e560-s004.tiff]
