# Supplementary material for: Prevalence and incidence of diabetic retinopathy (DR) in the UK population of Gloucestershire
Source: Acta Ophthalmol. 2021 Jun 28;100(2):e560–70. doi: 10.1111/aos.14927 (PMC9290830; doi:10.1111/aos.14927)
Supplement: Supplementary file 2 — Table S1. Diabetic Retinopathy Classifications of Progression to Proliferative DR. [file AOS-100-e560-s001.docx]

**Supplementary Table 1:** Diabetic Retinopathy Classifications of Progression to Proliferative DR

| ETDRS final Retinopathy Severity Scale | ETDRS  (Final)  Grade | Lesions | Risk of progression to PDR in 1 year  (ETDRS Interim) | ‘International’ Clinical Classification of Diabetic Retinopathy Severity | English Screening Programme levels |
| --- | --- | --- | --- | --- | --- |
| No apparent retinopathy | 10  14, 15 | DR absent  DR questionable |  |  | R0  Currently screen  Annually |
| Mild NPDR | 20 | Micro aneurysms only |  | Ma’s only | R1  Screen annually  Background  microaneurysm(s)  Retinal haemorrhage(s) ± any exudate  Venous loop |
|  | 35  a  b  c  d  e | One or more of the following:  Venous loops > definite in 1 field  SE, IRMA, or VB questionable  Retinal haemorrhages present  HE > definite in 1 field  SE > definite in 1 field | Level 30 = 6.2% | More than just micro aneurysms but less severe than Severe NPDR |  |
| Moderate NPDR | 43a  b | H/Ma moderate in 4-5 fields or severe in 1 field or  IRMA definite in 1-3 fields | Level 41 = 11.3% |  | R2  Refer to ophthalmologist  Pre-proliferative  venous beading  venous reduplication  intraretinal microvascular abnormality (IRMA  multiple deep, round or blot haemorrhages |
| Moderately severe NPDR | 47  a  b  c  d | Both level 43 characteristics –  H/Ma moderate in 4-5 fields or severe in 1 field and IRMA definite in 1-3 fields  or any one of the following:  IRMA in 4-5 fields  HMA severe in 2-3 fields  VB definite in 1 field | Level 45 = 20.7% | Severe NPDR  Any of the following:  a) Extensive intraretinal haem (>20) in 4 quadrants  b) Definite venous beading in 2+ quadrants  c) Prominent IRMA in 1+ quadrant  And no signs of PDR |  |
| Severe NPDR | 53  a  b  c  d | One or more of the following:  > 2 of the 3 level 47 characteristics  H/Ma severe in 4-5 fields  IRMA > moderate in 1 field  VB > definite in 2-3 fields | Level 51 = 44.2%  Level 55 = 54.8% |  |  |
| Mild PDR | 61a  b | FPD or FPE present with NVD absent or  NVE = definite | Neovascularisation  Vitreous / preretinal haemorrhage | | R3  Urgent referral to ophthalmologist Proliferative new vessels on disc (NVD)  new vessels elsewhere (NVE)  pre-retinal or vitreous haemorrhage  pre-retinal fibrosis ± tractional retinal detachment |
| Moderate PDR | 65a  b | 1. NVE > moderate in 1 field or definite NVD with VH and PRH absent or questionable or 2. VH or PRH definite and NVE < moderate in 1 field and NVD absent |  |  |  |
| High risk PDR | 71  a  b  c  d | Any of the following:   1. VH or PRH > moderate in 1 field 2. NVE > moderate in 1 field and VH or PRH definite in 1 field 3. NVD = 2 and VH or PRH definite in 1 field 4. NVD > moderate |  |  |  |
| High risk PDR | 75 | NVD > moderate and definite VH or PRH |  |  |  |
| Advanced PDR | 81 | Retina obscured due to VH or PRH |  |  |  |
| Abbreviations: ETDRS, *Early Treatment Diabetic Retinopathy Study*; DR, *diabetic retinopathy*; PDR, *proliferative DR*; Ma, *microaneurysm*; NPDR, *non-proliferative DR*; SE, *soft exudate*; IRMA, *intraretinal microvascular abnormality*; VB, *venous beading;* HE, *hard exudate*; H/Ma, *Haemorrhage/microaneurysm*; FPD, *fibrous proliferation on disc*; FPE, *fibrous proliferation elsewhere*; NVD, *new vessels on disc*; NVE, *new vessels elsewhere*; PRH, *pre-retinal haemorrhage*; VH, *Vitreous haemorrhage*. | | | | | |
