# Supplementary material for: Prevalence and incidence of diabetic retinopathy (DR) in the UK population of Gloucestershire
Source: Acta Ophthalmol. 2021 Jun 28;100(2):e560–70. doi: 10.1111/aos.14927 (PMC9290830; doi:10.1111/aos.14927)
Supplement: Supplementary file 3 — Table S2. Baseline characteristics of the subjects within the separate analysis cohorts. [file AOS-100-e560-s003.docx]

**Supplementary Table 2:** Baseline characteristics of the subjects within the separate analysis cohorts

| Characteristic | | Prevalence (n=35,873) | Incidence of DR (n=18,367) | Incidence of moderate NPDR or worse (n=27,023) | Incidence of PDR (n=28,178) | Survival analysis (n=404) |
| --- | --- | --- | --- | --- | --- | --- |
| Gender n (%*) | Recorded, n | 35,870 | 18,376 | 27,023 | 28,178 | 404 |
|  | Female | 15,346 (42.8) | 7,968 (43.4) | 11,580 (42.9) | 12,013 (42.6) | 200 (40.2) |
|  | Male | 20,524 (57.2) | 10,399 (56.6) | 15,443 (57.1) | 16,165 (57.4) | 297 (59.8) |
| Age (years) | Median (IQR) | 66 (56 – 75) | 66 (57 – 75) | 66 (57 – 75) | 66 (56 – 75) | 64 (54 – 72) |
|  | Mean (SD) | 64.6 (14.2) | 65.3 (13.0) | 65.1 (13.4) | 64.9 (13.5) | 62.2 (14.1) |
| Ethnicity n (%*) | Recorded, n | 21,988 | 10,795 | 16,641 | 17,786 | 393 |
|  | Caucasian | 20,767 (94.4) | 10,254 (95.0) | 15,782 (94.8) | 16,846 (94.7) | 366 (93.1) |
|  | Asian | 744 (3.4) | 352 (3.3) | 543 (3.3) | 585 (3.3) | 18 (4.5) |
|  | Black | 264 (1.2) | 105 (1.0) | 169 (1.0) | 195 (1.1) | 4 (1.0) |
|  | Mixed | 133 (0.6) | 51 (0.5) | 96 (0.6) | 105 (0.6) | 3 (0.8) |
|  | Other | 80 (0.4) | 33 (0.3) | 51 (0.3) | 55 (0.3) | 2 (0.5) |
| Diabetes Type n (%*) | Recorded, n | 35,681 | 18,354 | 27,007 | 28,160 | 404 |
|  | T1DM | 2,579 (7.2) | 542 (3.0) | 1,503 (5.6) | 1,803 (6.4) | 67 (16.6) |
|  | T2DM | 33,085 (92.7) | 17,806 (97.0) | 25,497 (94.4) | 26,349 (93.6) | 337 (83.4) |
|  | Other | 17 (0.05) | 6 (0.03) | 7 (0.03) | 8 (0.03) | 0 |
| Years since diabetes diagnosis** | Median (IQR) | 5 (1 - 10) | 4 (1 – 8) | 5 (2 – 10) | 5 (2 – 10) | 13 (8 – 18) |
|  | Mean (SD) | 7.4 (8.3) | 5.5 (5.6) | 7.0 (1.4) | 7.5 (7.8) | 14.0 (8.6) |
| Years since T1DM diagnosis** | Median (IQR) | 18 (8 – 31) | 6 (2 – 14) | 16 (7 – 27) | 17 (8 – 29) | 20 (16 – 29) |
|  | Mean (SD) | 20.4 (14.8) | 10.4 (12.0) | 18.4 (14.3) | 19.6 (14.2) | 22.6 (11.6) |
| Years since T2DM diagnosis** | Median (IQR) | 5 (1 – 9) | 4 (1 – 8) | 5 (2 – 9) | 5 (2 – 10) | 12 (7 – 17) |
|  | Mean (SD) | 6.4 (6.7) | 5.3 (5.2) | 6.4 (6.1) | 6.6 (6.4) | 12.2 (6.8) |
| HbA_1c_ (mmol/mol) n (%*) | Recorded, n | 26,358 | 14,247 | 20,837 | 21,682 | 329 |
|  | < 48 | 7,500 (28.5) | 4,825 (33.9) | 6,236 (29.9) | 6,296 (29.0) | 21 (6.4) |
|  | 48-57 | 7,977 (30.3) | 4,523 (31.8) | 6,368 (30.6) | 6,508 (30.0) | 51 (15.5) |
|  | 58-85 | 8,457 (32.1) | 3,961 (27.8) | 6,611 (31.7) | 7,047 (32.5) | 161 (48.9) |
|  | ≥ 86 | 2,424 (9.2) | 938 (6.6) | 1,622 (7.8) | 1,831 (8.4) | 96 (29.2) |
|  | Median (IQR) | 54 (47 – 67) | 52 (45 – 63) | 54 (47 – 65) | 54 (47 – 66) | 70 (59 – 91) |
|  | Mean (SD) | 59.4 (18.2) | 56.6 (16.7) | 58.4 (17.3) | 59.0 (17.7) | 75.9 (23.2) |
| Baseline severity of retinopathy in worse eye n (%*) | Recorded, n | 35,873 | 18,367 | 27,023 | 28,178 | 404 |
|  | None | 23,245 (64.8) | n/a | 18,367 (68.0) | 18,367 (65.2) | 37 (9.2) |
|  | Mild NPDR | 10,447 (29.1) |  | 8,656 (32.0) | 8,656 (30.7) | 367 (90.8) |
|  | Moderate-severe NPDR | 1,280 (3.6) |  | n/a | 1,155 (4.1) | n/a |
|  | Proliferative | 901 (2.5) |  |  | n/a |  |
| Abbreviations: T1DM, *Type 1 diabetes mellitus*; T2DM, *Type 2 diabetes mellitus*; GDESP, *Gloucestershire Diabetic Eye Screening Programme;* DR, *diabetic retinopathy;* NPDR, *non-proliferative DR*  Baseline was first complete assessment (under HEC, screening or surveillance) within the study period.  * Percentage was calculated using ‘recorded’ column n’s as the denominator.  ** Date of diagnosis of diabetes was not available for everyone. For 351 (1.0%), 68 (0.4%), 106 (0.4%), 121 (0.4%), and 5 (1.0%) people with diabetes, date of registration to the GDESP was used instead (with respect to each analysis cohort). | | | | | | |
