# Supplementary material for: Prevalence and incidence of diabetic retinopathy (DR) in the UK population of Gloucestershire
Source: Acta Ophthalmol. 2021 Jun 28;100(2):e560–70. doi: 10.1111/aos.14927 (PMC9290830; doi:10.1111/aos.14927)
Supplement: Supplementary file 4 — Table S3. Prevalence of moderate‐severe NPDR by ETDRS level, diabetes type, and calendar year, per 100 people with diabetes. [file AOS-100-e560-s005.docx]

**Supplementary Table 3:** Prevalence of moderate-severe NPDR by ETDRS level, diabetes type, and calendar year, per 100 people with diabetes

|  | | | **2012** | **2013** | **2014** | **2015** | **2016** | **IRR (95% CI) for trend over time (increment of calendar year)** |
| --- | --- | --- | --- | --- | --- | --- | --- | --- |
| Number of Gloucestershire PWD with one or more DR assessments during the respective year (denominator) | | Overall | 21,487 | 22,004 | 22,746 | 24,967 | 26,669 |  |
|  |  | T1DM | 1,555 | 1,514 | 1,578 | 1,809 | 1,911 |  |
|  |  | T2DM | 19,919 | 20,463 | 21,086 | 23,096 | 24,673 |  |
| Any DR (n) | | | 13,118 | 13,678 | 14,493 | 15,925 | 16,906 |  |
| Moderate-severe NPDR (n) | | | 981 | 841 | 809 | 907 | 835 |  |
| DESP R2 referrals ^a^ | | | 188 | 104 | 90 | 74 | 97 |  |
| ETDRS level 43 (Moderate NPDR) | Overall | n (%^b^) | 430 (54.2) | 405 (55.0) | 420 (58.4) | 528 (63.4) | 471 (63.8) | 0.99 (0.96 to 1.02) p=0.460 |
|  |  | Prevalence  (95% CI) | 2.0  (1.8 to 2.2) | 1.8  (1.7 to 2.0) | 1.8  (1.7 to 2.0) | 2.1  (1.9 to 2.3) | 1.8  (1.6 to 1.9) |  |
|  | T1DM | n | 94 | 90 | 104 | 139 | 112 | 1.02 (0.96 to 1.08) p=0.544 |
|  |  | Prevalence  (95% CI) | 6.0 (4.9 to 7.4) | 5.9 (4.8 to 7.3) | 6.6 (5.4 to 8.0) | 7.7 (6.5 to 9.1) | 5.9 (4.9 to 7.1) |  |
|  | T2DM | n | 336 | 315 | 316 | 388 | 359 | 0.98 (0.95 to 1.01) p=0.224 |
|  |  | Prevalence  (95% CI) | 1.7 (1.5 to 1.9) | 1.5 (1.4 to 1.7) | 1.5 (1.3 to 1.7) | 1.7 (1.5 to 1.9) | 1.5 (1.3 to 1.6) |  |
| ETDRS level 47 (Moderately severe NPDR) | Overall | n (%^b^) | 96 (12.1) | 90 (12.2) | 79 (11.0) | 93 (11.2) | 78 (10.6) | 0.91 (0.85 to 0.97) p=0.006 |
|  |  | Prevalence  (95% CI) | 0.45  (0.37 to 0.55) | 0.41  (0.33 to 0.50) | 0.35  (0.28 to 0.43) | 0.37  (0.30 to 0.46) | 0.29  (0.23 to 0.37) |  |
|  | T1DM | n | 13 | 13 | 12 | 15 | 14 | 0.97 (0.82 to 1.15) p=0.728 |
|  |  | Prevalence  (95% CI) | 0.84 (0.49 to 1.4) | 0.86 (0.50 to 1.5) | 0.76 (0.43 to 1.3) | 0.83 (0.50 to 1.4) | 0.73 (0.43 to 1.2) |  |
|  | T2DM | n | 82 | 76 | 66 | 77 | 63 | 0.90 (0.84 to 0.97) p=0.004 |
|  |  | Prevalence  (95% CI) | 0.41 (0.33 to 0.51) | 0.37 (0.30 to 0.47) | 0.31 (0.25 to 0.40) | 0.33 (0.27 to 0.42) | 0.26 (0.20 to 0.33) |  |
| ETDRS 53 (Severe NPDR) | Overall | n (% ^b^) | 267 (33.7) | 242 (32.8) | 220 (30.6) | 212 (25.5) | 189 (25.6) | 0.87 (0.84 to 0.91) p<0.001 |
|  |  | Prevalence  (95% CI) | 1.2  (1.1 to 1.4) | 1.1  (1.0 to 1.2) | 1.0  (0.85 to 1.1) | 0.85  (0.74 to 1.0) | 0.71  (0.61 to 0.82) |  |
|  | T1DM | n | 46 | 44 | 39 | 33 | 44 | 0.91 (0.83 to 1.0) p=0.053 |
|  |  | Prevalence  (95% CI) | 3.0 (2.2 to 4.0) | 2.9 (2.2 to 3.9) | 2.5 (1.8 to 3.4) | 1.8 (1.3 to 2.6) | 2.3 (1.7 to 3.1) |  |
|  | T2DM | n | 221 | 198 | 181 | 179 | 145 | 0.86 (0.83 to 0.90) p<0.001 |
|  |  | Prevalence  (95% CI) | 1.1 (1.0 to 1.3) | 0.97 (0.84 to 1.1) | 0.86 (0.74 to 1.0) | 0.78 (0.67 to 0.90) | 0.59 (0.50 to 0.69) |  |
| PWD, *people with diabetes*; DR, *diabetic retinopathy*; NPDR, *non-proliferative* DR; CI, *confidence interval*; ETDRS, *Early Treatment Diabetic Retinopathy Study*; DESP, *Diabetic Eye Screening Programme*; T1DM, *Type 1 diabetes mellitus*; T2DM, *Type 2 diabetes mellitus;* IRR, *incidence rate ratio*.  Prevalence was estimated using Poisson regression, where the denominator was the number of Gloucestershire PWD with at least one assessment during the respective year. For each PWD, their DR severity recorded for the respective year was based on the worst grade given to the worst eye that year. Trend over time was calculated by adding calendar year to the Poisson regression model. Overall also includes those with ‘other’ and ‘unknown’ type of diabetes.  ^a^ Those who were referred as having moderate-severe NPDR by the screening programme (who do not use ETDRS levels) but were not yet seen by HEC by the end of the calendar year.  ^b^ Percentage of patients with moderate-severe NPDR in at least one eye (whilst under HEC). | | | | | | | | |
