# Supplementary material for: Prevalence and incidence of diabetic retinopathy (DR) in the UK population of Gloucestershire
Source: Acta Ophthalmol. 2021 Jun 28;100(2):e560–70. doi: 10.1111/aos.14927 (PMC9290830; doi:10.1111/aos.14927)
Supplement: Supplementary file 5 — Table S4. Sensitivity analysis ‐ prevalence of DR by severity and calendar year, per 100 people with diabetes (regardless of appointment attendance). [file AOS-100-e560-s007.docx]

**Supplementary Table 4:** Sensitivity analysis - prevalence of DR by severity and calendar year, per 100 people with diabetes (regardless of appointment attendance)

|  | | **2012** | **2013** | **2014** | **2015** | **2016** | **IRR (95% CI) for trend over time (increment of calendar year)** |
| --- | --- | --- | --- | --- | --- | --- | --- |
| Number of Gloucestershire PWD on the diabetes register during the respective year *regardless of appointment attendance* (denominator) | | 30,314 | 30,882 | 31,541 | 32,051 | 34,065 |  |
| Any DR in at least one eye | n | 8,369 | 8,326 | 8,253 | 9,042 | 9,763 | 1.01 (1.00 to 1.02) p<0.001 |
|  | Prevalence  (95% CI) | 27.6  (27.0 to 28.2) | 27.0  (26.4 to 27.5) | 26.2  (25.6 to 26.7) | 28.2  (27.6 to 28.8) | 28.7  (28.1 to 29.2) |  |
| Mild NPDR in worst eye (ETDRS 20-35) | n (%^a^) | 6,630 (79.2) | 6,783 (81.5) | 6,781 (82.2) | 7,356 (81.4) | 8,113 (83.1) | 1.02 (1.01 to 1.03) p<0.001 |
|  | Prevalence  (95% CI) | 21.9  (21.4 to 22.4) | 22.0  (21.4 to 22.5) | 21.5  (21.0 to 22.0) | 23.0  (22.4 to 23.5) | 23.8  (23.3 to 24.3) |  |
| Moderate-severe NPDR in worst eye (ETDRS 43-53)^b^ | n (% ^a^) | 981 (11.7) | 841 (10.1) | 809 (9.8) | 907 (10.0) | 835 (8.6) | 0.95 (0.93 to 0.97) p<0.001 |
|  | Prevalence  (95% CI) | 3.2  (3.0 to 3.5) | 2.7  (2.6 to 2.9) | 2.6  (2.4 to 2.8) | 2.8  (2.7 to 3.0) | 2.5  (2.3 to 2.6) |  |
| Moderate NPDR  (ETDRS 43) | n (% ^a^) | 430 (5.1) | 405 (4.9) | 420 (5.1) | 528 (5.8) | 471 (4.8) | 1.02 (0.99 to 1.05) p=0.224 |
|  | Prevalence  (95% CI) | 1.4 (1.3 to 1.6) | 1.3 (1.2 to 1.4) | 1.3 (1.2 to 1.5) | 1.6 (1.5 to 1.8) | 1.4 (1.3 to 1.5) |  |
| Moderately severe  NPDR (ETDRS 47) | n (% ^a^) | 96 (1.1) | 90 (1.1) | 79 (1.0) | 93 (1.0) | 78 (0.8) | 0.94 (0.88 to 1.00) p=0.056 |
|  | Prevalence  (95% CI) | 0.32 (0.26 to 0.39) | 0.29 (0.24 to 0.36) | 0.25 (0.20 to 0.31) | 0.29 (0.24 to 0.36) | 0.23 (0.18 to 0.29) |  |
| Severe NPDR  (ETDRS 53) | n (% ^a^) | 267 (3.2) | 242 (2.9) | 220 (2.7) | 212 (2.3) | 189 (1.9) | 0.90 (0.86 to 0.93) p<0.001 |
|  | Prevalence  (95% CI) | 0.88 (0.78 to 0.99) | 0.78 (0.69 to 0.89) | 0.70 (0.61 to 0.80) | 0.66 (0.58 to 0.76) | 0.55 (0.48 to 0.64) |  |
| PDR in worst eye (ETDRS ≥ 61) | n (% ^a^) | 758 (9.1) | 702 (8.4) | 663 (8.0) | 779 (8.6) | 815 (8.3) | 1.00 (0.98 to 1.02) p=0.891 |
|  | Prevalence  (95% CI) | 2.5  (2.3 to 2.7) | 2.3  (2.1 to 2.5) | 2.1  (2.0 to 2.3) | 2.4  (2.3 to 2.6) | 2.4  (2.2 to 2.6) |  |
| PWD, *people with diabetes*; DR, *diabetic retinopathy*; NPDR, *non-proliferative* DR; PDR, *proliferative DR*; CI, *confidence interval*; IRR, *incidence rate ratio*.  Prevalence was estimated using Poisson regression, where the denominator was the number of people with diabetes, estimated by the number of people on the Gloucestershire Diabetic Eye Screening Programme register at each year midpoint, regardless of attendance. For each PWD, their DR severity recorded for the respective year was based on the worst grade given to the worst eye that year. Trend over time was calculated by adding calendar year to the Poisson regression model.  ^a^ Percentage of those with any DR in at least one eye; any DR is the denominator.  ^b^ Includes those who were referred as having moderate-severe NPDR by the screening programme (who do not use ETDRS levels) but were not yet seen by HEC by the end of the calendar year. | | | | | | | |
