# Supplementary material for: Prevalence and incidence of diabetic retinopathy (DR) in the UK population of Gloucestershire
Source: Acta Ophthalmol. 2021 Jun 28;100(2):e560–70. doi: 10.1111/aos.14927 (PMC9290830; doi:10.1111/aos.14927)
Supplement: Supplementary file 6 — Table S5. Unadjusted risk factors from univariable models of prevalence and incidence of any DR, moderate‐severe NPDR and proliferative DR. [file AOS-100-e560-s009.docx]

**Supplementary Table 5**: Unadjusted risk factors from univariable models of prevalence and incidence of any DR, moderate-severe NPDR and proliferative DR

|  | Any DR | | | Moderate-severe NPDR* | | | PDR | | |
| --- | --- | --- | --- | --- | --- | --- | --- | --- | --- |
|  | **Change in risk** | **95% CI** | **p-value** | **Change in risk** | **95% CI** | **p-value** | **Change in risk** | **95% CI** | **p-value** |
| **Risk Factor for Prevalence** |  |  |  |  |  |  |  |  |  |
| Time since diagnosis of diabetes (per 5 years) | +21% | 20% to 21% | <0.001 | +33% | 32% to 34% | <0.001 | +48% | 47% to 49% | <0.001 |
| HbA_1c_ (per 10 mmol/mol) | +15% | 15% to 16% | <0.001 | +38% | 36% to 39% | <0.001 | +37% | 35% to 39% | <0.001 |
| Gender: male | +5% | 3% to 7% | <0.001 | +12% | 6% to 19% | <0.001 | +23% | 15% to 31% | <0.001 |
| Diabetes type:  T1DM vs T2DM | +120% | 114% to 126% | <0.001 | +273% | 248% to 301% | <0.001 | +920% | 855% to 988% | <0.001 |
| Age (per 5 years) | -2% | -3% to -2% | <0.001 | -9% | -10% to -8% | <0.001 | -14% | -15% to -13% | <0.001 |
|  |  |  |  |  |  |  |  |  |  |
|  |  |  |  |  |  |  |  |  |  |
| **Risk Factor for Incidence** |  |  |  |  |  |  |  |  |  |
| Time since diagnosis of diabetes (per 5 years) | +20% | 18% to 22% | <0.001 | +34% | 31% to 38% | <0.001 | +39% | 35% to 44% | <0.001 |
| HbA_1c_ (per 10 mmol/mol) | +7% | 6% to 9% | <0.001 | +37% | 33% to 42% | <0.001 | +44% | 37% to 51% | <0.001 |
| Gender: male | -7% | -12% to -2% | 0.007 | +4% | -12% to +22% | 0.648 | +18% | -7% to +51% | 0.177 |
| Diabetes type:  T1DM vs T2DM | +26% | 10% to 44% | 0.001 | +286% | 214% to 374% | <0.001 | +553% | 404% to 746% | <0.001 |
| Age (per 5 years) | +5% | 4% to 6% | <0.001 | -10% | -12% to -8% | <0.001 | -17% | -20% to -13% | <0.001 |
| Abbreviations: DR, *diabetic retinopathy*; PDR, *proliferative DR*; IRR, *incidence rate ratio*; CI, *confidence interval*; T1DM, *Type 1 diabetes mellitus*; T2DM, *Type 2 diabetes mellitus*.  * For incidence, this was moderate NPDR or worse.  Age, time since diagnosis of diabetes, and HbA_1c_ were all measured at baseline (date of incident case or year midpoint for incidence and prevalence respectively) and categorised into 5 year and 10 mmol/mol groups.  Estimates of the effect of risk factors were estimated by adding each factor to the Poisson regression models of prevalence/incidence individually. Change in risk was calculated from the resulting IRRs ((estimate-1)*100). | | | | | | | | | |
