# Supplementary material for: Prevalence and incidence of diabetic retinopathy (DR) in the UK population of Gloucestershire
Source: Acta Ophthalmol. 2021 Jun 28;100(2):e560–70. doi: 10.1111/aos.14927 (PMC9290830; doi:10.1111/aos.14927)
Supplement: Supplementary file 7 — Table S6. Incidence of PDR by DR severity (no DR/mild NPDR or moderate‐severe NPDR) and diabetes type, per 100 people with diabetes. [file AOS-100-e560-s006.docx]

**Supplementary Table 6:** Incidence of PDR by DR severity (no DR/mild NPDR or moderate-severe NPDR) and diabetes type, per 100 people with diabetes

|  | | | **4-year [2013-2016]** | **2013** | **2014** | **2015** | **2016** |
| --- | --- | --- | --- | --- | --- | --- | --- |
| Number of Gloucestershire PWD with two or more DR assessments where the second or later assessment was *during the respective year/time period* | | | 28,976 | 17,774 | 20,173 | 22,372 | 23,263 |
| Number with no DR or mild NPDR prior (denominator) | | Overall | 16,401 | 23,173 | 24,963 | 26,378 | 28,588 |
|  |  | T1DM | 899 | 1,247 | 1,390 | 1,467 | 1,543 |
|  |  | T2DM | 15,499 | 21,909 | 23,499 | 24,817 | 26,915 |
| Developed new PDR (ETDRS ≥ 61) | Overall | n | 51 | 9 | 16 | 23 | 22 |
|  |  | Incidence (95% CI) | 0.31 (0.23 to 0.41) | 0.039 (0.018 to 0.074) | 0.064 (0.037 to 0.10) | 0.087 (0.055 to 0.13) | 0.077 (0.048 to 0.12) |
|  | T1DM | n | 15 | 2 | 4 | 6 | 8 |
|  |  | Incidence (95% CI) | 1.7 (0.93 to 2.8) | 0.16 (0.019 to 0.58) | 0.29 (0.078 to 0.74) | 0.41 (0.15 to 0.89) | 0.52 (0.22 to 1.0) |
|  | T2DM | n | 36 | 7 | 12 | 17 | 14 |
|  |  | Incidence (95% CI) | 0.23 (0.16 to 0.32) | 0.032 (0.013 to 0.066) | 0.051 (0.026 to 0.089) | 0.069 (0.040 to 0.11) | 0.052 (0.028 to 0.087) |
| Number with moderate-severe NPDR prior (denominator) | | Overall | 731 | 1,050 | 1,147 | 1,264 | 1,297 |
|  |  | T1DM | 193 | 247 | 264 | 303 | 310 |
|  |  | T2DM | 536 | 801 | 881 | 958 | 985 |
| Developed new PDR (ETDRS ≥ 61) | Overall | n | 156 | 55 | 41 | 46 | 57 |
|  |  | Incidence (95% CI) | 21.3 (18.1 to 25.0) | 5.2 (3.9 to 6.8) | 3.6 (2.6 to 4.8) | 3.6 (2.7 to 4.9) | 4.4 (3.3 to 5.7) |
|  | T1DM | n | 42 | 16 | 10 | 17 | 19 |
|  |  | Incidence (95% CI) | 21.8 (15.7 to 29.4) | 6.5 (3.7 to 10.5) | 3.8 (1.8 to 7.0) | 5.6 (3.3 to 9.0) | 6.1 (3.7 to 9.6) |
|  | T2DM | n | 114 | 39 | 31 | 29 | 38 |
|  |  | Incidence (95% CI) | 21.3 (17.5 to 25.6) | 4.9 (3.5 to 6.7) | 3.5 (2.4 to 5.0) | 3.0 (2.0 to 4.3) | 3.9 (2.7 to 5.3) |
| Abbreviation: PWD, *people with diabetes*; DR, *diabetic retinopathy*; PDR, *proliferative DR*; CI, *confidence interval*; T1DM, *Type 1 diabetes mellitus*; T2DM, Type 2 diabetes *mellitus*.  Incidence was estimated using Poisson regression, where the denominator was Gloucestershire PWD at risk. People counted towards the numerator if they had a record of the disease with a previous record of no disease. Incidence could not be estimated for calendar year 2012 due to the requirement of needing a prior record of no DR and subjects normally being seen annually. Overall also includes those with ‘other’ and ‘unknown’ type of diabetes. For 4-year incidence, subjects had to also be alive, registered and living in the area for the entire 4-year period to ensure complete follow-up. See Methods for further details | | | | | | | |
