# Supplementary material for: Prevalence and incidence of diabetic retinopathy (DR) in the UK population of Gloucestershire
Source: Acta Ophthalmol. 2021 Jun 28;100(2):e560–70. doi: 10.1111/aos.14927 (PMC9290830; doi:10.1111/aos.14927)
Supplement: Supplementary file 8 — Table S7. Risk factors for developing PDR from incident moderate‐severe NPDR (n patients = 404). [file AOS-100-e560-s002.docx]

**Supplementary Table 7:** Risk factors for developing PDR from incident moderate-severe NPDR (*n patients=404*)

| **Univariable models** | | | |
| --- | --- | --- | --- |
| Risk factor | Increment | Summary (median (IQR)) | Change in risk (95% CI) |
| T1DM | vs T2DM | 83.4% T2DM | +10% (-59% to +189%) |
| Female | vs male | 40.8% Female | +82% (-15% to 286%) |
| Age | 5 years | 66 years (55 - 75) | -12% (-22% to +1%) |
| Time since diagnosis of diabetes* | 5 years | 15 years (10 - 20) | +5% (-14% to +29%) |
| Updated mean HbA_1c_ ** | 10 mmol/mol | 69 mmol/mol (58 - 84) | **+23% (+7% to +40%)** |
| Alkaline phosphatase | 10 U/L | 79 U/L (64 - 99) | -3% (-16% to +12%) |
| ALT | 5 U/L | 20 U/L (15 – 27) | -6% (-22% to +13%) |
| Total Cholesterol | 1 mmol/l | 4.2 mmol/l (3.6 – 5.0) | +10% (-21% to +53%) |
| HDL | 1 mmol/l | 1.2 mmol/l (1.0 – 1.5) | -28% (-70% to +76%) |
| Potassium | 1 mmol/l | 4.6 mmol/l (4.3 – 4.9) | -23% (-67% to +81%) |
| Total Bilirubin | 1 µmol/L | 7 µmol/L (5 – 10) | -1% (-10% to +9%) |
| Total protein | 5 g/L | 69 g/L (67 – 72) | -3% (-36% to +46%) |
| Albumin / Creatinine ratio | 0.1 g/µmol | 0.43 g/µmol (0.34 – 0.54) | -4% (-26% to +25%) |
| Both eyes with moderate-severe NPDR at incidence case | vs one eye | 36.1% both eyes | **+163% (+20% to +474%)** |
| **Multi-variable model** | | | |
| Risk factor | Increment | Change in risk (95% CI) | |
| Updated mean HbA_1c_ ** | 10 mmol/mol | +18% (+3% to +35%) | |
| Both eyes with moderate-severe NPDR at incident case | vs one eye | +123% (+0%*** to +400%) | |
| Abbreviations: DR, *diabetic retinopathy*; PDR, *proliferative DR*; NPDR, *non-proliferative DR*; CI, *confidence interval*; T1DM, *Type 1 diabetes mellitus*; T2DM, *Type 2 diabetes mellitus*; ALT, *Alanine transaminase*; HDL, *High density lipoprotein*.  All continuous variables were taken at time of incident moderate-severe NPDR.  * Date of diagnosis of diabetes was not available for everyone, for 5 (1.2%) people with diabetes date of registration to the GDESP was used instead. ** Updated mean HbA1c is the weighted mean of current and previous HbA1c records (up to start of study period).  Weibull survival models were used. In the multi-variable model, variables were entered into the model using forward stepwise selection. Variables considered were all those in the univariable analyses. The change in risk values in the table were calculated from the resulting hazard ratios ((estimate-1)*100). Bolded values for univariable models were statistically significant.  ***The likelihood ratio test indicated that the inclusion of this variable significantly improved the model fit; p=0.048". | | | |
