# Supplementary material for: Prevalence and incidence of diabetic retinopathy (DR) in the UK population of Gloucestershire
Source: Acta Ophthalmol. 2021 Jun 28;100(2):e560–70. doi: 10.1111/aos.14927 (PMC9290830; doi:10.1111/aos.14927)
Supplement: Supplementary file 9 — Table S8. Number of participants in the denominator for incidence of any DR each calendar year. [file AOS-100-e560-s008.docx]

**Supplementary Table 8**: Number of participants in the denominator for incidence of any DR each calendar year

| **Year** | **Total denominator** | **Number of those that didn’t move onto consecutive denominator (i.e. the following year)** | **Number of those that were new to the denominator (i.e. not present the previous year)** |
| --- | --- | --- | --- |
| 2013 | 15,505 | 2,187 (14%) | n/a |
| 2014 | 15,341 | 1,977 (13%) | 2,023 (13%) |
| 2015 | 15,283 | 1,901 (12%) | 1,919 (13%) |
| 2016 | 16,111 | n/a | 2,729 (17%) |
